# Dynamics of Biomarkers in COVID-19 Patients Treated with Anakinra

**DOI:** 10.3390/biomedicines12122690

**Published:** 2024-11-25

**Authors:** Ralitsa Yordanova, Dimitar Strashimirov, Rusina Grozdeva, Daniel Ivanov, Ivelina Trifonova, Nina Yancheva, Tatiana Tcherveniakova

**Affiliations:** 1 Department of Infectious Diseases, Parasitology and Tropical Medicine, Medical University Sofia, 1431 Sofia, Bulgaria; dstrashimirov@yahoo.com (D.S.); rusina.s.grozdeva@gmail.com (R.G.); daniel.ivanov.md@gmail.com (D.I.); dr.yancheva@abv.bg (N.Y.); 2 National Laboratory “Influenza and ARD”, Department of Virology, National Center of Infectious and Parasitic Diseases (NCIPD), 1504 Sofia, Bulgaria; trifonova.ivelina@abv.bg

**Keywords:** COVID-19, hyperinflammation, treatment evaluation, IL-1 receptor antagonist

## Abstract

**Background:** SARS-CoV-2 can trigger hyperinflammation, leading to severe COVID-19, presenting with pneumonia, acute respiratory distress syndrome (ARDS), and multiple organ failure. Specific biomarkers like leukocytes, CRP, NLR, AST, LDH, D-dimer, ferritin, and IL-6 are associated with disease severity. Anakinra, an IL-1 receptor antagonist, has been proposed to mitigate hyperinflammation, but its clinical efficacy remains uncertain. This study aimed to evaluate the effect of Anakinra on inflammatory biomarkers, oxygenation status, and survival outcomes in hospitalized patients with moderate to severe COVID-19 (according to the National Institute of Health severity scale), compared to standard treatment. **Methods:** A retrospective analysis included 65 patients (mean age 75.51 ± 9.54 years; 58.5% male, 41.5% female) hospitalized with moderate to severe COVID-19. Patients were divided into two groups: a control group receiving standard treatment (n = 24) and a target group treated with Anakinra (n = 41). Biomarkers and oxygenation status were assessed on Days 0, 3, and 7. Statistical analyses compared the groups for changes in leukocytes, NLR, CRP, AST, LDH, D-dimer, ferritin, and IL-6. **Results:** Anakinra treatment was associated with significant reductions in leukocytes, NLR, D-dimer, ferritin, IL-6, and CRP by Days 3 and 7. Improvements in oxygenation status were observed, although no survival benefits were noted. The control group showed no significant biomarker changes except for AST and LDH on Day 7. **Conclusions:** Anakinra demonstrated favorable effects on biomarkers and oxygenation in moderate to severe COVID-19 but did not improve survival. Further studies are needed to validate these findings.

## 1. Introduction

The SARS-CoV-2 pandemic posed a significant challenge to the medical community. Although the infection typically leads to mild to moderate disease, with viral replication generally confined to the upper respiratory tract, severe to critical illness can sometimes develop. These clinical forms of disease often manifest as pneumonia, respiratory failure, and multiple organ failure. These forms typically develop about a week after the initial symptoms, with dyspnea being one of the most observed clinical manifestations, resulting from hypoxemia [1,2], followed by the progression to respiratory failure. Numerous studies have demonstrated the significance of dysregulated immune processes due to SARS-CoV-2 infection in the developing severe and critical COVID-19. The hyperactivation of immune cells (macrophages, NK cells, B and T lymphocytes) leads to abnormal systemic inflammation, with cytokine release syndrome (CRS), which has been identified as a pathogenic mechanism in the progression of severe COVID-19 [3,4,5,6].

Additionally, macrophage activation syndrome (MAS) has been observed, commonly seen in autoimmune and malignant diseases [7,8,9,10,11], as well as in sepsis. This syndrome is characterized by the hyperactivation of tissue macrophages and the overproduction of inflammatory cytokines, such as IL-1, IL-6, and IL-8, and elevated levels of markers of inflammation, C-reactive protein (CRP), ferritin, and coagulation disorders involving D-dimer [12,13,14,15,16,17,18,19,20,21]. Dysregulation in immune function is also reflected in the leucocyte number, showing leukocytosis, lymphopenia, and an increased neutrophil-to-lymphocyte ratio (NLR) [22,23], with a correlation established between lymphopenia and an elevated NLR on one side, and the severity of COVID-19 on the other. Numerous studies have been conducted on the dynamics of various biomarkers throughout SARS-CoV-2 infection. Changes in specific biomarkers have been identified as strong indicators of the degree of immune dysfunction, and the severity of infection, and have high prognostic value for the outcome of the disease. These include elevated ferritin levels (a result of macrophage activation), increased NLR, elevated levels of aspartate aminotransferase (ASAT) and lactate dehydrogenase (LDH), indicative of liver involvement, increased D-dimer due to coagulation abnormalities, and elevated CRP and IL-6, indicative of hyperinflammation [24,25,26,27,28].

The dysregulation of immune function in severe and critical clinical forms of COVID-19 provides a rationale for including medications in the treatment regimen that modulate the immune response through various mechanisms.

In 2021, the European Medicines Agency (EMA), and in 2022, the U.S. Food and Drug Administration (FDA) approved the use of Anakinra for COVID-19 [29,30,31]. Anakinra is a recombinant human IL-1 receptor antagonist that binds to IL-1α and IL-1β receptors. Studies have demonstrated a connection between the early initiation of Anakinra therapy in hospitalized COVID-19 patients and the positive dynamics of examined biomarkers, including the reduction in CRP, ferritin, D-dimer, and NLR [32,33,34,35,36,37].

Considering the above, a study was conducted to evaluate the dynamics of biomarkers in the treatment of hospitalized patients with moderate to severe COVID-19 using Anakinra, compared to those receiving standard care.

## 2. Materials and Methods

### 2.1. Study Population

A retrospective clinical–epidemiological study was conducted, involving 65 patients with moderate to severe clinical forms of SARS-CoV-2 infection (according to the National Institute of Health NIH severity scale) who were hospitalized at the Specialized Hospital for Active Treatment of Infectious and Parasitic Diseases “Prof. Ivan Kirov”, Sofia with COVID-19 during the period from November 2022 to November 2023.

To enhance the internal validity of the study and improve the statistical power of detected associations within this selected patient cohort, the study carefully defines the cohort using precise inclusion and exclusion criteria. This approach reduces confounding factors and variability in the sample, thereby allowing for more robust results.

The following inclusion criteria were used: positive PCR test for SARS-CoV-2, age over 18 years, hospital admission between the 5th and 10th day from disease onset, radiological evidence of pneumonia, presence of at least 5 of the following laboratory criteria: WBC > 10.5 × 10^9^/L, NLR > 5.0, CRP > 60 mg/L, ASAT > 38 U/L, LDH > 250 U/L, D-dimer > 0.5 mg/L, ferritin > 250 ng/mL, IL-6 > 25 pg/mL.

The exclusion criteria were as follows: age under 18 years, pregnant women, patients with COVID-19 who are hospitalized for reasons other than COVID-19, patients with CLcr < 50 mL/min, patients who refuse to sign informed consent on the day of admission, patients transferred to ICU, patients who could not complete 7 days of Anakinra treatment or whose hospital stay was less than 7 days.

All data used in the study were collected from the medical records of patients who signed an informed consent form for the use of their data for scientific purposes on the day of hospital admission. The study was approved by the ethics committee at Specialized Hospital for Active Treatment of Infectious and Parasitic Diseases “Prof. Ivan Kirov”, Sofia (protocol number 14/2022).

### 2.2. Specimen Sampling and Biomarker Measurement

The diagnosis of COVID-19 was etiologically confirmed by qualitatively detecting the genetic material of SARS-CoV-2 in samples taken from epithelial cells of the upper respiratory tract (nasopharyngeal swabs). A multiplex qualitative RT–PCR (Real-Time Polymerase Chain Reaction) analysis was used to detect the viral RNA (SARS-CoV-2 RNA).

The PCR diagnostic system for SARS-CoV-2 contains specific oligonucleotide primers, fluorescently labeled probes targeting specific regions of the viral genes, and the matrix RNA (mRNA) of human ribonuclease P. The system also included the reverse transcriptase enzyme (DNA polymerase), a human ribonuclease inhibitor, free deoxynucleotides (dNTPs), and a positive control. The reaction proceeded through the following steps: 1. Extraction of viral RNA; 2. Reverse transcription of complementary DNA; 3. Amplification of specific sections of complementary DNA using specific oligonucleotide primers, the reverse transcriptase enzyme, and free deoxynucleotides as building blocks of complementary DNA; and 4. Visualization of the PCR products (amplicons).

The PCR diagnostic system simultaneously detected the following three different regions of the SARS-CoV-2 viral genome: the Nucleocapsid gene (N), the Envelope gene (E), and the RNA-dependent RNA polymerase gene (RdRP), as well as the matrix RNA (mRNA) of human ribonuclease P (RNase P). The detection of RNase P mRNA was an internal control to monitor the RNA extraction and amplification processes, reducing false-negative results.

In addition to the internal control, the RT–PCR reaction included positive and negative controls. For each target gene, the reaction was considered positive if the fluorescence curve for the corresponding gene crossed the threshold line by the 40^th^ cycle (Ct < 40). The result was considered positive if at least two of the three SARS-CoV-2 genes tested positive. If only one of the three target genes was positive, the result was considered indeterminate, and a new test was required (Table 1).

The analysis was conducted using the Tianlong^®^ Real-Time PCR System (Tianlong Technology Co., Xi’an, China). Reagents from the SOLIScript^®^ SARS-CoV-2 RT-qPCR Multiplex Assay by Solis BioDyne (Tartu, Estonia) were used for the RT–PCR analysis of SARS-CoV-2 RNA. These reagents comply with ISO 9001 [38] and ISO 13485 [39] standards.

For the patients included in the study, hematological (WBC, NLR), biochemical (CRP, ASAT, LDH, ferritin), coagulation (D-dimer), and immunological (IL-6) parameters were measured on days 1, 3, and 7. The tests were conducted in the Clinical Laboratory of Specialized Hospital for Active Treatment of Infectious and Parasitic Diseases “Prof. Ivan Kirov”, Sofia, which is accredited according to ISO 9001 [38] and SGS.

Blood samples for testing the parameters in blood and serum were collected using vacutainer tubes—closed vacuum tubes. Hematological parameters (WBC, neutrophils, lymphocytes) were determined using the automated hematology analyzer Arcus 380 (Diatron Group, Budapest, Hungary). Whole blood collected with EDTA anticoagulant was used for this analysis. The analysis is based on the impedance method. For biochemical (CRP, ASAT, LDH, ferritin) and immunological (IL-6) parameter measurements, vacutainer tubes with a clot activator, which accelerates the blood clotting process and separates serum needed for the analysis, were used. The analyses were conducted on the Selectra pro S automated biochemical analyzer (ELITechGroup, Puteaux, France), while IL-6 was measured using the Cobas e 411 automated analyzer (Roche Diagnostics, Basel, Switzerland). Plasma was used as the sample material for D-dimer testing. Vacutainer tubes containing sodium citrate were used, and the testing was performed on the Finecare Fia Meter Wondfo (Guangzhou Wondfo Biotech Co., Guangzhou, China).

The analysis of CRP is based on the immunofluorescent method, and ASAT is analyzed using the method recommended by the International Federation of Clinical Chemistry (IFCC). The LDH is analyzed through a chemical reaction involving the oxidation of lactate to pyruvate. Ferritin is measured using an immunoturbidimetric assay, D-dimer by fluorescent immunoassay, and IL-6 by chemiluminescence immunoassay.

The measurements of hematological parameters (WBC, neutrophils, lymphocytes) and biochemical parameters (CRP, ASAT, LDH, ferritin, and D-dimer) were conducted immediately after sample collection. Following centrifugation of the blood samples, the separated sera for the IL-6 measurement were stored at −80 °C until analysis.

### 2.3. Statistical Methods

Data were entered and processed using the statistical software packages IBM SPSS Statistics 25.0 (IBM Corp., Armonk, NY, USA), MedCalc Version 19.6.3 (www.medcalc.org; accessed 22 November 2024), and Excel Office 2021 (Microsoft Corp., Redmond, WA, USA). Differences with *p*-values less than 0.05 were considered statistically significant.

The following statistical methods were applied: descriptive analysis–frequency distribution of the observed variables was presented in tabular form; graphical analysis was used for visualizing the obtained results; Comparison of Relative Shares; Fisher’s Exact Test, Fisher–Freeman–Halton Exact Test, and Chi-square χ2 for testing the hypothesis for dependency between categorical variables; Kolmogorov–Smirnov and Shapiro–Wilk Nonparametric Tests were used to test the normality of distributions. Student’s T-Test was used to test hypotheses regarding the differences between the means of two independent samples; the Mann–Whitney Nonparametric Test was used to test hypotheses regarding the differences between two independent samples; repeated measures ANOVA–analysis of variance–was used to compare arithmetic averages in multiple comparisons; Mauchly’s Test was performed to check the assumptions of sphericity in repeated measures ANOVA; the Friedman Nonparametric Test was used to test hypotheses regarding differences between multiple dependent samples; and the Wilcoxon Nonparametric Test was used to test hypotheses regarding differences between two dependent samples.

## 3. Results

### 3.1. Characteristics of the Group

The average age of the study population was 75.51 ± 9.54 years, ranging from 44 to 99 years. Among the participants in the study sample, 38 (58.5%) were men and 27 (41.5%) were women.

**Figure 1:**
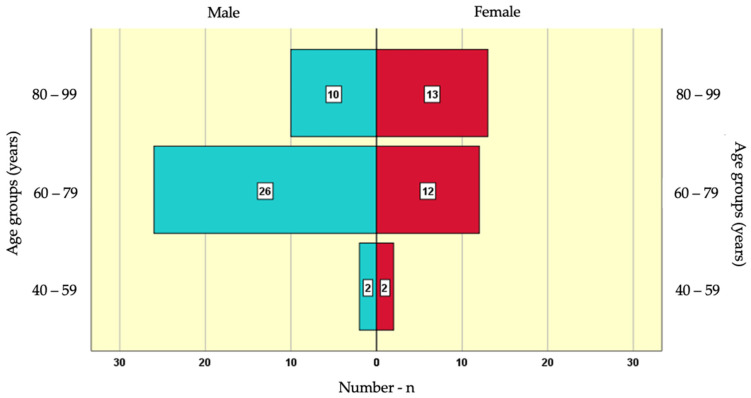
Distribution of Study Participants by Gender and Age Groups.

The age group with the highest number of male participants (26) was 60–79 years, followed by 80–99 years with 10 participants, and the smallest group (2) was 40–59 years. Among female participants, the largest age group (13) was 80–99 years, followed by 60–79 years with 12 participants, and the smallest group (2) was 40–59 years (Figure 1).

For this study, the patients were divided into the following two groups:

Control Group (Standard treatment)-n =24 (36.9%)

Target Group (Anakinra treatment)-n = 41 (63.1%)

Both groups received anticoagulant treatment. Patients requiring additional oxygen were provided with conventional oxygen supplementation.

General Characteristics of the Groups.

The two study groups were statistically balanced regarding the key confounding factors of gender and age, which is a strong basis for the accuracy of subsequent comparisons. The control and target groups did not show statistical differences in the following variables: clinical form, presence of chronic diseases, risk factors, antibiotic treatment, dexamethasone administration, duration of oxygen supplementation, duration of hospitalization, and 28-day survival rate (Table 2 and Table 3).

In the following Table 4, Table 5, Table 6, Table 7, Table 8, Table 9, Table 10, Table 11, Table 12, Table 13, Table 14, Table 15, Table 16, Table 17, Table 18, Table 19, Table 20, Table 21, Table 22 and Table 23, the grey color highlights the differences between measurements taken on Day 3 and Day 7 compared to the baseline values (both in absolute terms and percentages) where the dynamics are statistically significant. The yellow color indicates higher values in comparisons between male and female groups, as well as across different age groups.

### 3.2. Dynamics of WBC

#### 3.2.1. Dynamics of WBC in the Control Group

No statistically significant dynamic changes in WBC values were observed for the entire sample or in the subgroups formed based on gender and age. Additionally, no statistically significant differences were observed between the subgroups at any of the three control time points (Table 4).

**Table 4:** Dynamics of WBC by Gender and Age in the Control Group.

Indicator	Group	n	Day of the Examination					
			1		3		7	
			$\bar{X}$	SD	$\bar{X}$	SD	$\bar{X}$	SD
	Total	24	7.92 ^a^	4.23	8.26 ^a^	3.69	8.60 ^a^	4.34
Difference	(absolute)				0.34	3.06	0.68	4.01
Difference	%				21.4	92.4	27.4	102.2
	Male	15	8.10 ^a^	4.04	8.29 ^a^	4.04	8.91 ^a^	4.81
Difference	(absolute)				0.18	1.35	0.81	2.42
Difference	%				4.3	15.1	11.4	29.7
	Female	9	7.61 ^a^	4.77	8.21 ^a^	3.26	8.06 ^a^	3.63
Difference	(absolute)				0.60	4.85	0.45	5.99
Difference	%				50.0	150.6	54.0	165.0
	*p* =		0.428		0.815		0.861	
Age Group	60–79	13	7.86 ^a^	4.19	8.11 ^a^	4.26	8.42 ^a^	4.42
Difference	(absolute)				0.25	1.45	0.56	2.19
Difference	%				5.4	17.0	11.0	30.6
Age Group	80–99	9	8.71 ^a^	4.68	9.06 ^a^	3.03	9.36 ^a^	4.75
Difference	(absolute)				0.35	4.86	0.65	6.22
Difference	%				45.3	151.8	50.1	166.1
	*p* =		0.662		0.292		0.695	

Identical letters across the horizontal rows indicate no statistically significant difference, while different letters indicate the presence of a statistically significant difference (*p* < 0.05). Changes are measured on Day 3 and Day 7, compared to the baseline values on Day 1.

#### 3.2.2. Dynamics of WBC in the Target Group

A statistically significant decrease in WBC values was observed on Day 3 for the entire sample, as well as in the male subgroup. The absolute decrease ranged from 1 to 3 units; the percentage decrease was between −15% and −32%. No significant differences were observed between the subgroups (Table 5).

**Table 5:** Dynamics of WBC by Gender and Age in the Target Group.

Indicator	Group	n	Day of the Examination					
			1		3		7	
			$\bar{X}$	SD	$\bar{X}$	SD	$\bar{X}$	SD
	Total	41	7.23 ^a^	3.9	5.69 ^bc^	2.80	6.02 ^ac^	2.35
Difference	(absolute)				−1.53	3.28	−1.21	3.87
Difference	%				−14.9	38.5	−5.2	44.6
	Male	23	7.39 ^a^	3.64	5.65 ^bc^	3.19	5.93 ^ac^	2.47
Difference	(absolute)				−1.74	3.69	−1.46	4.20
Difference	%				−18.8	39.2	−9.5	40.7
	Female	18	7.02 ^a^	2.86	5.75 ^a^	2.29	6.13 ^a^	2.27
Difference	(absolute)				−1.27	2.74	−0.88	3.51
Difference	%				−9.8	38.1	0.3	49.8
	*p* =		0.813		0.478		0.655	
Age Group	60–79	25	7.30 ^a^	2.94	6.19 ^a^	3.16	6.49 ^a^	2.41
Difference	(absolute)				−1.11	3.12	−0.80	3.34
Difference	%				−11.7	36.5	−2.3	40.2
Age Group	80–99	14	7.51 ^a^	3.99	5.07 ^a^	2.07	5.50 ^a^	2.18
Difference	(absolute)				−2.44	3.69	−2.01	4.94
Difference	%				−21.0	45.0	−9.2	55.1
	*p* =		0.919		0.361		0.195	

Identical letters across the horizontal rows indicate no statistically significant difference, while different letters indicate the presence of a statistically significant difference (*p* < 0.05). Changes are measured on Day 3 and Day 7, compared to the baseline values on Day 1. The grey color highlights the differences between measurements taken on Day 3 and Day 7 compared to the baseline values (both in absolute terms and percentages) where the dynamics are statistically significant.

### 3.3. Dynamics of NLR

#### 3.3.1. Dynamics of NLR in the Control Group

The only statistically significant dynamic change was observed on Day 7, both in the entire sample and in the 60–79 age group. On this day, the measured values were significantly lower by around 3–4 units in absolute terms, and the percentage decrease ranged from 1% in the entire sample to 27% in the younger age group compared to baseline values. No statistically significant differences were found between the subgroups at any of the control checks (Table 6).

**Table 6:** Dynamics of NLR by Gender and Age in the Control Group.

Indicator	Group	N	Day of the Examination					
			1		3		7	
			$\bar{X}$	SD	$\bar{X}$	SD	$\bar{X}$	SD
	Total	24	8.27 ^a^	7.04	6.70 ^a^	5.82	5.49 ^b^	4.39
Difference	(absolute)				−1.58	5.08	−2.78	6.24
Difference	%				−3.1	61.9	−1.3	95.2
	Male	15	7.21	5.11	6.73 ^a^	6.40	5.21 ^a^	4.61
Difference	(absolute)				−0.48	3.87	−2.00	4.97
Difference	%				−9.3	44.2	−11.2	81.0
	Female	9	10.04 ^a^	9.54	6.64 ^a^	5.08	5.95 ^a^	4.21
Difference	(absolute)				−3.40	6.47	−4.09	8.09
Difference	%				7.1	86.1	15.3	118.6
	*p* =		0.428		0.318		0.411	
Age Group	60–79	13	8.84 ^a^	5.44	6.99 ^a^	5.93	4.97 ^b^	3.50
Difference	(absolute)				−1.85	3.49	−3.87	4.73
Difference	%				−23.5	30.7	−27.0	80.3
Age Group	80–99	9	8.85 ^a^	9.34	7.05 ^a^	6.38	6.44 ^a^	5.77
Difference	(absolute)				−1.80	7.31	−2.41	8.22
Difference	%				14.5	86.3	12.0	99.5
	*p* =		0.695		0.948		0.744	

Identical letters across the horizontal rows indicate no statistically significant difference, while different letters indicate the presence of a statistically significant difference (*p* < 0.05). Changes are measured on Day 3 and Day 7, compared to the baseline values on Day 1. The grey color highlights the differences between measurements taken on Day 3 and Day 7 compared to the baseline values (both in absolute terms and percentages) where the dynamics are statistically significant.

#### 3.3.2. Dynamics of NLR in the Target Group

In this group, the dynamics of NLR were much more pronounced (Table 7) as follows:

- Day 3: A significant decrease was observed in the entire sample, as well as in the male subgroup, female subgroup, and the 80–99 age group.
- Day 7: A statistically significant decrease was seen in the entire sample, as well as in the male subgroup, female subgroup, and the 60–79 age group.
- In absolute terms, the significant decrease ranged from 3 to 6 units, while the percentage decrease ranged from 11% to 45%.
- Day 3: Statistically significant differences (or borderline significance, *p* < 0.1) were observed between male and female subgroups (with higher average values in men) and between age groups (with higher average values in younger patients).
- Day 7: No significant differences were found between the subgroups.

**Table 7:** Dynamics of NLR by Gender and Age in the Target Group.

Indicator	Group	N	Day of the Examination					
			1		3		7	
			$\bar{X}$	SD	$\bar{X}$	SD	$\bar{X}$	SD
	Total	41	8.97 ^a^	7.71	5.01 ^b^	5.91	4.26 ^b^	4.50
Difference	(absolute)				−3.97	7.83	−4.71	8.36
Difference	%				−26.7	66.2	−16.3	112.4
	Male	23	10.60 ^a^	8.84	6.09 ^b^	6.89	4.95 ^b^	4.79
Difference	(absolute)				−4.51	9.76	−5.65	9.47
Difference	%				−23.1	73.4	−20.0	95.8
	Female	18	6.89 ^a^	5.53	3.62 ^b^	4.13	3.39 ^b^	4.07
Difference	(absolute)				−3.28	4.47	−3.51	6.75
Difference	%				−31.2	57.5	−11.6	133.5
	*p* =		0.172		0.062		0.217	
Age Group	60–79	25	9.69 ^a^	8.15	6.46 ^ac^	7.04	4.72 ^bc^	4.51
Difference	(absolute)				−3.23	8.42	−4.97	8.18
Difference	%				−18.0	59.3	−20.6	73.6
Age Group	80–99	14	8.38 ^a^	7.44	2.85 ^bc^	2.26	3.86 ^ac^	4.79
Difference	(absolute)				−5.53	7.31	−4.52	9.52
Difference	%				−38.7	80.9	−1.2	167.9
	*p* =		0.534		0.044		0.149	

Identical letters across the horizontal rows indicate no statistically significant difference, while different letters indicate the presence of a statistically significant difference (*p* < 0.05). Changes are measured on Day 3 and Day 7, compared to the baseline values on Day 1. The grey color highlights the differences between measurements taken on Day 3 and Day 7 compared to the baseline values (both in absolute terms and percentages) where the dynamics are statistically significant. The yellow color indicates higher values in comparisons between male and female groups, as well as across different age groups.

### 3.4. Dynamics of CRP

#### 3.4.1. Dynamics of CRP in the Control Group

The following results from Table 8 show that a statistically significant decrease in CRP values was observed:

- Both control checks (Day 3 and Day 7): A significant reduction was observed in the entire sample and all subgroups.
- The absolute decrease ranged from 61 to 141 units, with a greater reduction on Day 7. In percentage terms, the decrease ranged from 37% to 73%.
- No significant differences were found between the subgroups.

**Table 8:** Dynamics of CRP by Gender and Age in the Control Group.

Indicator	Group	n	Day of the Examination					
			1		3		7	
			$\bar{X}$	SD	$\bar{X}$	SD	$\bar{X}$	SD
	Total	24	161.04 ^a^	103.51	87.75 ^b^	76.56	43.69 ^c^	42.28
Difference	(absolute)				−73.29	59.62	−117.35	90.67
Difference	%				−40.9	39.9	−60.5	69.2
	Male	15	176.07 ^a^	112.68	97.74 ^b^	87.90	40.28 ^c^	40.22
Difference	(absolute)				−78.32	64.07	−135.79	103.02
Difference	%				−37.6	46.9	−58.4	87.1
	Female	9	135.99 ^a^	86.36	71.09 ^b^	53.22	49.38 ^c^	47.44
Difference	(absolute)				−64.90	53.91	−86.61	57.97
Difference	%				−46.4	25.9	−64.0	22.2
	*p* =		0.238		0.379		0.770	
Age Group	60–79	13	188.06 ^a^	117.53	100.38 ^b^	92.78	46.78 ^c^	44.36
Difference	(absolute)				−87.68	71.07	−141.28	113.19
Difference	%				−37.4	51.2	−51.6	93.8
Age Group	80–99	9	140.90 ^a^	80.39	79.16 ^b^	55.84	44.51 ^c^	44.94
Difference	(absolute)				−61.74	40.21	−96.39	42.26
Difference	%				−46.8	22.9	−72.0	13.7
	*p* =		0.110		0.616		0.920	

Identical letters across the horizontal rows indicate no statistically significant difference, while different letters indicate the presence of a statistically significant difference (*p* < 0.05). Changes are measured on Day 3 and Day 7, compared to the baseline values on Day 1. The grey color highlights the differences between measurements taken on Day 3 and Day 7 compared to the baseline values (both in absolute terms and percentages) where the dynamics are statistically significant.

#### 3.4.2. Dynamics of CRP in the Target Group

From Table 9, it is evident that a statistically significant reduction in CRP values was observed as follows:

- Both control checks (Day 3 and Day 7): A significant reduction was seen in the entire sample and all subgroups.
- The absolute decrease ranged from 64 to 145 units, with a larger reduction on Day 7. In percentage terms, the decrease ranged from 48% to 92%.
- No significant differences were found between the subgroups.

**Table 9:** Dynamics of CRP by Gender and Age in the Target Group.

Indicator	Group	n	Day of the Examination					
			1		3		7	
			$\bar{X}$	SD	$\bar{X}$	SD	$\bar{X}$	SD
	Total	41	146.95 ^a^	54.34	68.12 ^b^	62.17	17.28 ^c^	24.96
Difference	(absolute)				−78.83	44.46	−129.67	38.84
Difference	%				−56.4	27.0	−89.8	9.2
	Male	23	147.79 ^a^	65.06	83.78 ^b^	76.57	22.13 ^c^	31.82
Difference	(absolute)				−64.01	46.05	−125.66	41.74
Difference	%				−48.3	30.7	−87.8	11.2
	Female	18	145.89 ^a^	38.31	48.12 ^b^	27.43	11.09 ^c^	9.11
Difference	(absolute)				−97.77	35.05	−134.79	35.28
Difference	%				−66.8	16.8	−92.4	5.1
	*p* =		0.694		0.156		0.415	
Age Group	60–79	25	144.01 ^a^	62.25	71.68 ^b^	71.40	17.22 ^c^	27.90
Difference	(absolute)				−72.33	46.99	−126.78	43.50
Difference	%				−54.0	29.1	−90.0	8.9
Age Group	80–99	14	155.18 ^a^	41.34	63.90 ^b^	47.67	18.16 ^c^	21.55
Difference	(absolute)				−91.27	41.74	−137.01	31.80
Difference	%				−60.2	24.6	−89.3	10.6
	*p* =		0.228		0.919		0.988	

Identical letters across the horizontal rows indicate no statistically significant difference, while different letters indicate the presence of a statistically significant difference (*p* < 0.05). Changes are measured on Day 3 and Day 7, compared to the baseline values on Day 1. The grey color highlights the differences between measurements taken on Day 3 and Day 7 compared to the baseline values (both in absolute terms and percentages) where the dynamics are statistically significant.

### 3.5. Dynamics of ASAT

#### 3.5.1. Dynamics of ASAT in the Control Group

From Table 10, a statistically significant decrease in ASAT values was observed as follows:

- Day 3: A significant reduction was seen in patients from the higher age subgroup.
- Day 7: A significant decrease was observed in the entire sample, as well as in patients from the higher age subgroup.
- The absolute decrease ranged from 4 to 18 units, with a larger reduction on Day 7. In percentage terms, the decrease ranged from 10% to 34%.
- No significant differences were observed between the subgroups.

**Table 10:** Dynamics of ASAT by Gender and Age in the Control Group.

Indicator	Group	n	Day of the Examination					
			1		3		7	
			$\bar{X}$	SD	$\bar{X}$	SD	$\bar{X}$	SD
	Total	24	44.08 ^a^	32.62	38.04 ^a^	31.12	31.83 ^b^	17.84
Difference	(absolute)				−6.04	15.24	−12.25	23.35
Difference	%				−10.6	32.6	−18.4	37.4
	Male	15	43.07 ^a^	36.41	41.93 ^a^	37.49	32.80 ^a^	18.98
Difference	(absolute)				−1.13	12.96	−10.27	23.09
Difference	%				−1.4	35.5	−15.6	23.3
	Female	9	45.78 ^a^	27.10	31.56 ^a^	15.76	30.22 ^a^	16.72
Difference	(absolute)				−14.22	15.92	−15.56	24.80
Difference	%				−26.0	20.9	−23.0	55.1
	*p* =		0.770		0.815		1.000	
Age Group	60–79	13	43.62 ^a^	38.51	42.00 ^a^	39.82	33.38 ^a^	18.91
Difference	(absolute)				−1.62	14.44	−10.23	26.96
Difference	%				−2.1	40.4	−9.7	46.1
Age Group	80–99	9	46.44 ^a^	26.43	32.78 ^b^	14.27	28.00 ^c^	12.20
Difference	(absolute)				−13.67	15.76	−18.44	19.06
Difference	%				−23.5	16.5	−33.7	17.1
	*p* =		0.695		0.896		0.462	

Identical letters across the horizontal rows indicate no statistically significant difference, while different letters indicate the presence of a statistically significant difference (*p* < 0.05). Changes are measured on Day 3 and Day 7, compared to the baseline values on Day 1. The grey color highlights the differences between measurements taken on Day 3 and Day 7 compared to the baseline values (both in absolute terms and percentages) where the dynamics are statistically significant.

#### 3.5.2. Dynamics of ASAT in the Target Group

From Table 11, it is evident that a statistically significant reduction in ASAT values was observed as follows:

- Day 3: A significant decrease was observed in the entire sample.
- Day 7: A significant reduction was seen in the entire sample, both genders, and both age subgroups.
- The absolute decrease ranged from 5 to 19 units, with a larger reduction on Day 7. In percentage terms, the decrease ranged from 4% to 30%.
- No significant differences were observed between the subgroups.

**Table 11:** Dynamics of ASAT by Gender and Age in the Target Group.

Indicator	Group	n	Day of the Examination					
			1		3		7	
			$\bar{X}$	SD	$\bar{X}$	SD	$\bar{X}$	SD
	Total	41	45.85 ^a^	26.71	41.07 ^b^	24.93	32.20 ^c^	16.42
Difference	(absolute)				−4.78	24.32	−13.66	25.62
Difference	%				−4.1	45.2	−20.3	37.0
	Male	23	45.43 ^a^	27.31	42.78 ^a^	24.24	34.17 ^b^	19.53
Difference	(absolute)				−2.65	20.31	−11.26	26.02
Difference	%				−1.0	33.5	−16.8	38.7
	Female	18	46.39 ^a^	26.69	38.89 ^a^	26.31	29.67 ^b^	11.33
Difference	(absolute)				−7.50	29.06	−16.72	25.49
Difference	%				−8.1	57.6	−24.7	35.3
	*p* =		0.554		0.554		0.772	
Age Group	60–79	25	43.12 ^a^	24.43	39.76 ^a^	24.11	31.12 ^b^	16.18
Difference	(absolute)				−3.36	18.36	−12.00	21.89
Difference	%				−4.6	36.4	−21.2	35.0
Age Group	80–99	14	44.00 ^a^	25.66	40.43 ^ac^	27.61	31.00 ^bc^	14.19
Difference	(absolute)				−3.57	31.08	−13.00	27.30
Difference	%				0.2	60.4	−16.9	41.1
	*p* =		0.874		0.828		0.942	

Identical letters across the horizontal rows indicate no statistically significant difference, while different letters indicate the presence of a statistically significant difference (*p* < 0.05). Changes are measured on Day 3 and Day 7, compared to the baseline values on Day 1. The grey color highlights the differences between measurements taken on Day 3 and Day 7 compared to the baseline values (both in absolute terms and percentages) where the dynamics are statistically significant.

### 3.6. Dynamics of LDH

#### 3.6.1. Dynamics of LDH in the Control Group

The results from Table 12 show that no statistically significant dynamic changes in LDH values were observed. Additionally, no significant differences were found between the subgroups.

**Table 12:** Dynamics of LDH by Gender and Age in the Control Group.

Indicator	Group	n	Day of the Examination					
			1		3		7	
			$\bar{X}$	SD	$\bar{X}$	SD	$\bar{X}$	SD
	Total	24	337.00 ^ac^	115.27	317.63 ^a^	122.88	284.46 ^bc^	137.08
Difference	(absolute)				−19.38	126.95	−52.54	140.56
Difference	%				−1.4	38.7	−12.4	32.4
	Male	15	312.80 ^a^	90.67	315.67 ^a^	110.31	277.40 ^a^	90.87
Difference	(absolute)				2.87	114.85	−35.40	97.95
Difference	%				5.7	44.3	−7.1	29.5
	Female	9	377.33 ^a^	144.53	320.89 ^a^	148.65	296.22 ^a^	198.30
Difference	(absolute)				−56.44	144.13	−81.11	196.32
Difference	%				−13.3	24.8	−21.2	36.8
	*p* =		0.190		0.861		0.815	
Age Group	60–79	13	310.62 ^a^	85.48	324.54 ^a^	110.29	286.92 ^a^	90.81
Difference	(absolute)				13.92	112.66	−23.69	95.89
Difference	%				8.8	45.6	−3.6	29.7
Age Group	80–99	9	371.44 ^a^	152.96	307.78 ^a^	153.15	278.00 ^a^	202.15
Difference	(absolute)				−63.67	152.33	−93.44	199.17
Difference	%				−14.0	28.5	−24.4	37.5
	*p* =		0.245		0.292		0.209	

Identical letters across the horizontal rows indicate no statistically significant difference, while different letters indicate the presence of a statistically significant difference (*p* < 0.05). Changes are measured on Day 3 and Day 7, compared to the baseline values on Day 1.

#### 3.6.2. Dynamics of LDH in the Target Group

Statistically significant reductions in LDH values were observed (Table 13) as follows:

- Day 3: A significant decrease was noted in the entire sample and the female subgroup.
- Day 7: A significant decrease was observed in the entire sample and both gender subgroups (male and female).
- The absolute decrease ranged between 40 and 105 units, with a higher reduction on Day 7. In percentage terms, the decrease ranged from 9% to 30%.
- No significant differences were observed between the subgroups.

**Table 13:** Dynamics of LDH by Gender and Age in the Target Group.

Indicator	Group	n	Day of the Examination					
			1		3		7	
			$\bar{X}$	SD	$\bar{X}$	SD	$\bar{X}$	SD
	Total	41	343.59 ^a^	139.06	302.93 ^b^	173.60	286.83 ^b^	209.91
Difference	(absolute)				−40.66	142.97	−56.76	171.43
Difference	%				−8.8	33.1	−16.8	38.9
	Male	23	331.65 ^a^	145.01	326.52 ^ac^	215.41	306.13 ^bc^	267.32
Difference	(absolute)				−5.13	150.89	−25.52	202.89
Difference	%				−1.4	35.8	−12.4	47.3
	Female	18	358.83 ^a^	133.59	272.78 ^b^	95.61	262.17 ^b^	100.23
Difference	(absolute)				−86.06	121.42	−96.67	113.45
Difference	%				−18.1	27.4	−22.5	24.8
	*p* =		0.331		0.723		0.713	
Age Group	60–79	25	332.40 ^a^	136.07	305.28 ^a^	141.47	276.72 ^a^	165.61
Difference	(absolute)				−27.12	127.62	−55.68	143.75
Difference	%				−3.4	33.7	−15.7	40.2
Age Group	80–99	14	358.79 ^a^	156.85	300.50 ^a^	234.50	308.29 ^a^	288.27
Difference	(absolute)				−58.29	177.23	−50.50	226.00
Difference	%				−16.2	33.2	−16.8	39.9
	*p* =		0.586		0.553		0.761	

Identical letters across the horizontal rows indicate no statistically significant difference, while different letters indicate the presence of a statistically significant difference (*p* < 0.05). Changes are measured on Day 3 and Day 7, compared to the baseline values on Day 1. The grey color highlights the differences between measurements taken on Day 3 and Day 7 compared to the baseline values (both in absolute terms and percentages) where the dynamics are statistically significant.

### 3.7. Dynamics of D-Dimer

#### 3.7.1. Dynamics of D-Dimer in the Control Group

Table 14 shows that no statistically significant dynamic changes were observed in the values of the examined parameter. However, a significant (or borderline statistically significant, *p* < 0.1) difference was found between the age subgroups, with higher average values observed in older patients.

**Table 14:** Dynamics of D-dimer by Gender and Age in the Control Group.

Indicator	Group	n	Day of the Examination					
			1		3		7	
			$\bar{X}$	SD	$\bar{X}$	SD	$\bar{X}$	SD
	Total	24	1.76 ^a^	1.59	1.87 ^a^	1.71	1.69 ^a^	1.80
Difference	(absolute)				0.12	1.13	−0.06	1.67
Difference	%				25.5	80.4	9.2	99.6
	Male	15	1.61 ^ac^	1.62	1.46 ^a^	1.30	1.10 ^bc^	0.97
Difference	(absolute)				−0.16	0.86	−0.52	1.04
Difference	%				20.1	80.2	−10.5	63.4
	Female	9	2.00 ^a^	1.59	2.56 ^a^	2.13	2.68 ^a^	2.44
Difference	(absolute)				0.57	1.43	0.69	2.26
Difference	%				34.4	84.7	42.0	139.9
	*p* =		0.482		0.238		0.138	
Age Group	60–79	13	1.60 ^a^	1.76	1.34 ^a^	1.39	1.14 ^a^	1.12
Difference	(absolute)				−0.27	0.86	−0.46	1.15
Difference	%				19.3	86.0	−7.4	69.0
Age Group	80–99	9	2.07 ^a^	1.54	2.62 ^a^	2.09	2.50 ^a^	2.49
Difference	(absolute)				0.54	1.43	0.43	2.34
Difference	%				28.1	84.2	29.6	142.5
	*p* =		0.209		0.082		0.186	

Identical letters across the horizontal rows indicate no statistically significant difference, while different letters indicate the presence of a statistically significant difference (*p* < 0.05). Changes are measured on Day 3 and Day 7, compared to the baseline values on Day 1. The yellow color indicates higher values in comparisons between male and female groups, as well as across different age groups.

#### 3.7.2. Dynamics of D-Dimer in the Target Group

A statistically significant reduction in the values of the examined parameter was observed (Table 15) as follows:

- Day 3: A significant decrease was noted in the entire sample and all subgroups, except for the 80–99 age group.
- Day 7: A significant decrease was observed in the entire sample and across all subgroups.
- The absolute decrease ranged from 0.19 to 1.03 units, with a higher reduction observed on Day 7. In percentage terms, the decrease ranged from 8% to 53%.
- A significant (or borderline statistically significant, *p* < 0.1) difference was found between the age subgroups, with higher average values observed in older patients.

**Table 15:** Dynamics of D-dimer by Gender and Age in the Target Group.

Indicator	Group	n	Day of the Examination					
			1		3		7	
			$\bar{X}$	SD	$\bar{X}$	SD	$\bar{X}$	SD
	Total	41	1.57 ^a^	1.58	1.34 ^b^	1.91	0.96 ^c^	1.87
Difference	(absolute)				−0.24	1.32	−0.61	2.23
Difference	%				−11.1	70.7	−26.8	97.8
	Male	23	1.88 ^a^	1.98	1.70 ^b^	2.44	1.24 ^b^	2.45
Difference	(absolute)				−0.19	1.75	−0.64	2.99
Difference	%				−8.3	83.2	−15.4	127.1
	Female	18	1.18 ^a^	0.74	0.87 ^b^	0.68	0.60 ^c^	0.46
Difference	(absolute)				−0.30	0.37	−0.58	0.44
Difference	%				−14.7	52.7	−41.4	34.5
	*p* =		0.581		0.783		0.743	
Age Group	60–79	25	1.29 ^a^	1.09	0.98 ^b^	1.01	0.86 ^b^	1.23
Difference	(absolute)				−0.32	0.89	−0.44	1.48
Difference	%				−13.6	72.1	−17.1	107.6
Age Group	80–99	14	2.12 ^a^	2.25	2.07 ^a^	2.91	1.24 ^b^	2.79
Difference	(absolute)				−0.05	1.96	−0.88	3.34
Difference	%				−1.6	73.9	−37.4	86.1
	*p* =		0.185		0.098		0.784	

Identical letters across the horizontal rows indicate no statistically significant difference, while different letters indicate the presence of a statistically significant difference (*p* < 0.05). Changes are measured on Day 3 and Day 7, compared to the baseline values on Day 1. The grey color highlights the differences between measurements taken on Day 3 and Day 7 compared to the baseline values (both in absolute terms and percentages) where the dynamics are statistically significant. The yellow color indicates higher values in comparisons between male and female groups, as well as across different age groups.

### 3.8. Dynamics of Ferritin

#### 3.8.1. Dynamics of Ferritin in the Control Group

No significant dynamic changes in ferritin levels were observed in the control group, or in the subgroups formed based on gender or age. Additionally, no statistically significant differences were found between the subgroups at any of the three time points during the control checks (Table 16).

**Table 16:** Dynamics of Ferritin by Gender and Age in the Control Group.

Indicator	Group	n	Day of the Examination					
			1		3		7	
			$\bar{X}$	SD	$\bar{X}$	SD	$\bar{X}$	SD
	Total	24	878.18 ^a^	749.52	832.09 ^a^	711.07	848.72 ^a^	883.93
Difference	(absolute)				−46.09	234.95	−29.45	525.89
Difference	%				4.4	40.2	4.5	56.5
	Male	15	974.59 ^a^	798.55	879.66 ^a^	746.24	944.46 ^a^	1002.22
Difference	(absolute)				−94.93	257.72	−30.13	626.42
Difference	%				0.4	42.0	1.1	53.7
	Female	9	717.48 ^a^	672.83	752.80 ^a^	683.96	689.16 ^a^	665.38
Difference	(absolute)				35.32	174.74	−28.32	329.23
Difference	%				11.2	38.6	10.1	63.9
	*p* =		0.347		0.770		519	
Age Group	60–79	13	1011.31 ^a^	857.90	933.26 ^a^	802.69	1037.60 ^a^	1067.67
Difference	(absolute)				−78.05	291.34	26.29	679.18
Difference	%				1.3	47.0	7.0	59.4
Age Group	80–99	9	696.37 ^a^	666.77	698.57 ^a^	670.53	647.72 ^a^	637.14
Difference	(absolute)				2.20	164.28	−48.64	278.76
Difference	%				11.4	35.3	10.0	58.3
	*p* =		0.357		0.556		0.512	

Identical letters across the horizontal rows indicate no statistically significant difference, while different letters indicate the presence of a statistically significant difference (*p* < 0.05). Changes are measured on Day 3 and Day 7, compared to the baseline values on Day 1.

#### 3.8.2. Dynamics of Ferritin in the Target Group

From Table 17, it is evident that a statistically significant reduction in ferritin values was observed as follows:

- Both control checks (Day 3 and Day 7): Significant decreases were observed in the entire sample and all subgroups.
- The absolute decrease ranged from 134 to 409 units, with a greater reduction noted on Day 7. In percentage terms, the decrease ranged from 11% to 40%.
- No significant differences were found between the subgroups.

**Table 17:** Dynamics of Ferritin by Gender and Age in the Target Group.

Indicator	Group	n	Day of the Examination					
			1		3		7	
			$\bar{X}$	SD	$\bar{X}$	SD	$\bar{X}$	SD
	Total	41	783.42 ^a^	740.26	597.65 ^b^	569.24	480.10 ^c^	422.31
Difference	(absolute)				−185.77	323.04	−303.31	503.67
Difference	%				−20.7	32.9	−29.1	43.5
	Male	23	804.03 ^a^	545.61	669.56 ^b^	525.60	583.96 ^b^	455.27
Difference	(absolute)				−134.48	245.20	−220.07	350.64
Difference	%				−15.4	38.3	−21.5	52.0
	Female	18	757.08 ^a^	950.17	505.77 ^b^	623.70	347.40 ^c^	343.74
Difference	(absolute)				−251.31	399.53	−409.68	645.33
Difference	%				−27.5	23.5	−38.7	27.9
	*p* =		0.207		0.207		0.098	
Age Group	60–79	25	835.43 ^a^	862.68	628.08 ^b^	642.91	499.52 ^c^	467.20
Difference	(absolute)				−207.35	385.75	−335.91	593.29
Difference	%				−20.0	38.5	−26.7	51.9
Age Group	80–99	14	711.04 ^a^	528.79	573.56 ^b^	471.95	446.24 ^c^	365.23
Difference	(absolute)				−137.48	183.62	−264.81	350.74
Difference	%				−21.1	22.4	−33.8	28.5
	*p* =		0.919		0.828		0.851	0.919

Identical letters across the horizontal rows indicate no statistically significant difference, while different letters indicate the presence of a statistically significant difference (*p* < 0.05). Changes are measured on Day 3 and Day 7, compared to the baseline values on Day 1. The grey color highlights the differences between measurements taken on Day 3 and Day 7 compared to the baseline values (both in absolute terms and percentages) where the dynamics are statistically significant.

### 3.9. Dynamics of IL-6

#### 3.9.1. Dynamics of IL-6 in the Control Group

No statistically significant dynamic changes in IL-6 values were observed in the control group, or in the subgroups formed based on gender and age. Additionally, no significant differences were found between the subgroups at any of the three time points during the control checks (Table 18).

**Table 18:** Dynamics of IL-6 by Gender and Age in the Control Group.

Indicator	Group	n	Day of the Examination					
			1		3		7	
			$\bar{X}$	SD	$\bar{X}$	SD	$\bar{X}$	SD
	Total	24	122.65 ^a^	303.71	62.99 ^a^	84.18	44.67 ^a^	37.06
Difference	(absolute)				−59.66	224.75	−77.98	291.72
Difference	%				−9.3	45.5	0.1	100.9
	Male	15	163.81 ^a^	381.74	74.07 ^a^	103.63	50.45 ^a^	38.94
Difference	(absolute)				−89.74	282.86	−113.36	367.59
Difference	%				−8.5	51.2	13.1	117.2
	Female	9	54.06 ^a^	41.23	44.54 ^a^	31.04	35.03 ^a^	33.58
Difference	(absolute)				−9.52	26.17	−19.03	44.09
Difference	%				−10.7	36.7	−21.7	66.4
	*p* =		0.640		0.726		0.318	
Age Group	60–79	13	177.91 ^a^	410.07	80.81 ^a^	109.74	46.81 ^a^	39.58
Difference	(absolute)				−97.10	303.91	−131.11	393.62
Difference	%				−5.4	49.0	11.8	125.7
Age Group	80–99	9	63.86 ^a^	43.10	45.87 ^a^	32.94	46.72 ^a^	38.02
Difference	(absolute)				−17.99	40.80	−17.13	47.14
Difference	%				−14.0	47.6	−11.6	70.6
	*p* =		0.896		0.471		0.948	

Identical letters across the horizontal rows indicate no statistically significant difference, while different letters indicate the presence of a statistically significant difference (*p* < 0.05). Changes are measured on Day 3 and Day 7, compared to the baseline values on Day 1.

#### 3.9.2. Dynamics of IL-6 in the Target Group

The results from Table 19 show that a statistically significant reduction in IL-6 values was observed as follows:

- Both control checks (Day 3 and Day 7): A significant decrease was observed in the entire sample and all subgroups.
- The absolute decrease ranged from 55 to 142 units, with a greater reduction on Day 7. In percentage terms, the decrease ranged from 50% to 75%.
- No significant differences were found between the subgroups.

**Table 19:** Dynamics of IL-6 by Gender and Age in the Target Group.

Indicator	Group	n	Day of the Examination					
			1		3		7	
			$\bar{X}$	SD	$\bar{X}$	SD	$\bar{X}$	SD
	Total	41	120.33 ^a^	184.10	34.90 ^b^	66.79	12.37 ^c^	22.64
Difference	(absolute)				−85.43	167.64	−107.96	186.07
Difference	%				−55.1	40.0	−65.9	61.4
	Male	23	109.90 ^a^	150.47	42.88 ^b^	79.62	15.99 ^c^	29.44
Difference	(absolute)				−67.02	121.63	−93.90	155.86
Difference	%				−54.5	40.2	−60.1	76.5
	Female	18	133.66 ^a^	223.86	24.71 ^b^	45.79	7.73 ^c^	6.66
Difference	(absolute)				−108.95	214.33	−125.93	222.31
Difference	%				−55.8	41.0	−73.3	34.4
	*p* =		0.528		0.331		0.237	
Age Group	60–79	25	139.86 ^a^	208.43	44.55 ^b^	80.73	13.33 ^c^	27.31
Difference	(absolute)				−95.30	185.46	−126.53	211.37
Difference	%				−57.0	35.9	−65.4	74.0
Age Group	80–99	14	98.53 ^a^	146.72	21.54 ^b^	34.75	11.76 ^c^	13.74
Difference	(absolute)				−76.99	148.43	−86.78	147.65
Difference	%				−50.4	49.2	−64.9	38.0
	*p* =		0.988		0.874		0.346	

Identical letters across the horizontal rows indicate no statistically significant difference, while different letters indicate the presence of a statistically significant difference (*p* < 0.05). Changes are measured on Day 3 and Day 7, compared to the baseline values on Day 1. The grey color highlights the differences between measurements taken on Day 3 and Day 7 compared to the baseline values (both in absolute terms and percentages) where the dynamics are statistically significant.

### 3.10. Dynamics of Oxygen Saturation

#### 3.10.1. Dynamics of Oxygen Saturation in the Control Group

- Statistically significant increases in oxygen saturation were observed on Day 7 in the entire group, the male subgroup, and the younger patient subgroup.
- The absolute increase ranged from 1 to 4 units, corresponding to a 1% to 4% increase in percentage terms.
- No statistically significant changes in oxygen saturation dynamics were observed across subgroups based on gender and age (see Table 20).

**Table 20:** Dynamics of oxygen saturation by Gender and Age in the Control Group.

Indicator	Group	n	Day of the Examination					
			1		3		7	
			$\bar{X}$	SD	$\bar{X}$	SD	$\bar{X}$	SD
	Total	24	91.33 ^a^	3.62	91.71 ^a^	4.45	93.50 ^b^	5.38
Difference	(absolute)				0.59	2.38	2.32	4.19
Difference	%				0.6	2.7	2.5	4.9
	Male	15	91.53 ^a^	3.04	92.13 ^a^	3.38	94.87 ^b^	1.51
Difference	(absolute)				0.93	1.94	3.57	2.21
Difference	%				1.0	2.2	4.0	2.6
	Female	9	91.00 ^a^	4.61	91.00 ^a^	6.00	91.22 ^a^	8.36
Difference	(absolute)				0.00	3.07	0.13	5.91
Difference	%				0.00	3.6	0.00	7.00
	*p* =		0.763		0.861		0.411	
Age Group	60–79	13	91.77 ^a^	3.27	92.38 ^a^	3.52	94.77 ^b^	1.69
Difference	(absolute)				0.62	1.76	3.00	2.08
Difference	%				0.7	2.0	3.3	2.4
Age Group	80–99	9	90.11 ^a^	4.20	90.67 ^a^	5.83	91.44 ^a^	8.41
Difference	(absolute)				0.56	3.21	1.33	6.14
Difference	%				0.6	3.7	1.4	7.2
	*p* =		0.292		0.471		0.324	

Identical letters across the horizontal rows indicate no statistically significant difference, while different letters indicate the presence of a statistically significant difference (*p* < 0.05). Changes are measured on Day 3 and Day 7, compared to the baseline values on Day 1. The grey color highlights the differences between measurements taken on Day 3 and Day 7 compared to the baseline values (both in absolute terms and percentages) where the dynamics are statistically significant.

#### 3.10.2. Dynamics of Oxygen Saturation in the Target Group

The results in Table 21 show a statistically significant increase in oxygen saturation as follows:

- For both control checks (Day 3 and Day 7), a significant increase was observed in the entire target group as well as in the subgroups.
- The absolute increase in oxygen saturation ranged from 1 to 5 units, with a greater increase noted on Day 7. In percentage terms, this increase ranged from 1% to 6%.
- On Day 3, there was a significant difference (or borderline significance) between the age subgroups, with a higher mean oxygen saturation observed in the older patient subgroup.

**Table 21:** Dynamics of oxygen saturation by Gender and Age in the Target Group.

Indicator	Group	n	Day of the Examination					
			1		3		7	
			$\bar{X}$	SD	$\bar{X}$	SD	$\bar{X}$	SD
	Total	41	91.02 ^a^	4.13	92.80 ^b^	4.05	94.56 ^c^	5.20
Difference	(absolute)				1.78	1.99	3.54	4.87
Difference	%				2.0	2.3	4.0	5.8
	Male	23	90.91 ^a^	4.58	92.39 ^b^	4.74	93.65 ^c^	6.77
Difference	(absolute)				1.48	2.37	2.74	5.89
Difference	%				1.7	2.7	3.1	7.0
	Female	18	91.17 ^a^	3.60	93.33 ^b^	3.01	95.72 ^c^	1.36
Difference	(absolute)				2.17	1.34	4.56	2.99
Difference	%				2.4	1.5	5.1	3.5
	*p* =		0.884		0.792		0.747	
Age Group	60–79	25	90.20 ^a^	4.26	92.08 ^b^	3.76	94.60 ^c^	4.19
Difference	(absolute)				1.88	1.86	4.40	4.71
Difference	%				2.1	2.2	5.0	5.6
Age Group	80–99	14	92.14 ^a^	3.88	93.71 ^b^	4.51	94.21 ^b^	7.07
Difference	(absolute)				1.57	2.38	2.07	5.31
Difference	%				1.7	2.7	2.2	6.2
	*p* =		0.195		0.098		0.289	

Identical letters across the horizontal rows indicate no statistically significant difference, while different letters indicate the presence of a statistically significant difference (*p* < 0.05). Changes are measured on Day 3 and Day 7, compared to the baseline values on Day 1. The grey color highlights the differences between measurements taken on Day 3 and Day 7 compared to the baseline values (both in absolute terms and percentages) where the dynamics are statistically significant. The yellow color indicates higher values in comparisons between male and female groups, as well as across different age groups.

## 4. Discussion

The relationship between the severity of COVID-19, patient outcomes, and the values and dynamics of biomarkers such as leukocytes, neutrophil-to-lymphocyte ratio (NLR), CRP, ASAT, LDH, D-dimers, ferritin, and IL-6 has been demonstrated in numerous studies.

In their study, Chaudhary et al. (2021) found significantly higher levels of CRP, D-dimers, ferritin, and IL-6 in patients with severe COVID-19 and those with fatal outcomes [14]. These results are consistent with those reported by Ullah et al. (2022), who, in a study of 500 COVID-19 patients, demonstrated a correlation between elevated levels of biomarkers, including CRP, LDH, D-dimers, and IL-6 with the severity of COVID-19 and the likelihood of a fatal outcome [40]. Similar conclusions were drawn by other authors as follows: Yun et al. (2020) observed significantly higher levels of neutrophils, CRP, ASAT, LDH, D-dimers, and lower levels of lymphocytes in patients with severe and critical forms of COVID-19 [17]. Elshazli et al. (2020), in a meta-analysis, also identified similar correlations between elevated biomarker levels, including increased leukocyte counts, and the progression to critical COVID-19 [18]. Wu et al. (2020) highlighted the importance of elevated neutrophil, LDH, and D-dimer levels as risk factors for the development of ARDS and death [16,41].

The hyperactivation of the immune system, resulting in abnormal inflammation and cytokine release syndrome, has been established as a pathogenic mechanism in the development of severe and critical COVID-19. The effect of medications that modulate the immune response on the above-mentioned biomarkers has been examined in multiple studies [5,33,42]. In this context, the role of Anakinra, an interleukin-1 receptor antagonist, has been explored in numerous studies [32,33,34,35,37,43].

In the present study, the values and dynamics of the indicated biomarkers were monitored in patients treated with Anakinra, compared to those who received standard treatment.

The analysis from Table 22 reveals the following findings regarding the dynamics of the studied blood parameters (WBC, NLR, and CRP):

- WBC–The dynamics of WBC significantly depend on the administration of Anakinra. In the Anakinra group, there was a statistically significant decrease in WBC values, whereas no dynamic changes were observed in the control group. Additionally, the Anakinra group showed significantly lower average WBC values during the control checks on Day 3 and Day 7.
- NLR (Neutrophil-to-Lymphocyte Ratio)–Average NLR values are significantly lower in the Anakinra group on Days 3 and 7 compared to the control group.
- CRP (C-reactive Protein)–The dynamics of CRP (specifically the downward trend) were not statistically significantly dependent on Anakinra administration. However, significantly lower average CRP values were observed in the Anakinra group during the control check on Day 7 compared to the control group.

**Table 22:** Dynamics of Blood Parameters (WBC, NLR, and CRP) by Group, Gender, and Age.

Indicator	Group	n	Day of the Examination					
			1		3		7	
			$\bar{X}$	SD	$\bar{X}$	SD	$\bar{X}$	SD
WBC	Control Group	24	7.92 ^a^	4.23	8.26 ^a^	3.69	8.60 ^a^	4.34
	Anakinra	41	7.23 ^ac^	3.29	5.69 ^b^	2.80	6.02 ^bc^	2.35
	*p* =		0.639		0.002		0.012	
NLR	Control Group	24	8.27 ^ad^	7.04	6.70 ^bd^	5.82	5.49 ^c^	4.39
	Anakinra	41	8.97 ^a^	7.71	5.01 ^b^	5.91	4.26 ^b^	4.50
	*p* =		0.755		0.033		0.075	
CRP	Control Group	24	161.04 ^a^	103.51	87.75 ^b^	76.56	43.69 ^c^	42.28
	Anakinra	41	146.95 ^a^	54.34	68.12 ^b^	62.17	17.28 ^c^	24.96
	*p* =		0.978		0.206		<0.001	

Identical letters across the horizontal rows indicate no statistically significant difference, while different letters indicate the presence of a statistically significant difference (*p* < 0.05). Changes are measured on Day 3 and Day 7, compared to the baseline values on Day 1. The grey color highlights the differences between measurements taken on Day 3 and Day 7 compared to the baseline values (both in absolute terms and percentages) where the dynamics are statistically significant. The yellow color indicates higher values in comparisons between male and female groups, as well as across different age groups.

From Table 23, the following observations are made regarding the dependence of the dynamics of the studied blood parameters (ASAT, LDH, D-dimer, ferritin, and IL-6) and oxygen saturation (Sat.O_2_):

- ASAT–the dynamics of ASAT are significantly influenced by Anakinra treatment. In the Anakinra group, there is a consistent and statistically significant decrease in ASAT values throughout the study, while in the control group, a statistically significant drop was only observed during the control check on Day 7.
- LDH–The dynamics of LDH also show a significant dependence on Anakinra treatment. In the Anakinra group, a statistically significant decrease occurred as early as Day 3. In the control group, a significant decrease was only observed on Day 7, and this was only when compared to Day 3, not the baseline values.
- D-dimer–The dynamics of D-dimer levels are significantly dependent on Anakinra treatment. The Anakinra group showed a consistent and statistically significant reduction in D-dimer levels, whereas no dynamics were observed in the control group. Furthermore, significantly (or borderline significantly, *p* < 0.1) lower mean D-dimer values were recorded in the Anakinra group on Days 3 and 7.
- Ferritin–There is a significant dependence of the downward trend in ferritin levels on Anakinra treatment. The Anakinra group experienced a consistent and statistically significant decrease in ferritin levels, while no significant changes were observed in the control group. Additionally, borderline significantly lower mean ferritin values were noted in the Anakinra group during the control checks on Days 3 and 7.
- IL-6–The downward trend in IL-6 levels is significantly influenced by Anakinra treatment. In the Anakinra group, a consistent and statistically significant decrease in IL-6 was observed throughout the study, while no significant changes were detected in the control group. Furthermore, significantly lower IL-6 values were recorded in the Anakinra group during the control checks on Days 3 and 7.
- Oxygen saturation–The upward trend of this indicator is statistically significantly influenced only by the administration of Anakinra and by age, as observed at the control check on Day 7. In the Anakinra group, there is a consistent statistically significant increase, whereas in the control group, the increase appears only on Day 7. In younger patients, the increase in the indicator’s values is consistent, while in older patients, the increase on Day 7 is statistically similar to that on Day 3. Additionally, a significantly higher mean value can be noted among patients receiving Anakinra at the control check on Day 7.

**Table 23:** Dynamics of Blood Parameters (ASAT, LDH, D-dimer, Ferritin, and IL-6) and oxygen saturation (Sat.O_2_).

Indicator	Group	n	Day of the Examination					
			1		3		7	
			$\bar{X}$	SD	$\bar{X}$	SD	$\bar{X}$	SD
ASAT	Control Group	24	44.08 ^a^	32.62	38.04 ^a^	31.12	31.83 ^b^	17.84
	Anakinra	41	45.85 ^a^	26.71	41.07 ^b^	24.93	32.20 ^c^	16.42
	*p* =		0.514		0.395		0.828	
LDH	Control Group	24	337.00 ^ac^	115.27	317.63 ^a^	122.88	284.46 ^bc^	137.08
	Anakinra	41	343.59 ^a^	139.06	302.93 ^b^	173.60	286.83 ^b^	209.91
	*p* =		0.930		0.135		0.324	
D-dimer	Control Group	24	1.76 ^a^	1.59	1.87 ^a^	1.71	1.69 ^a^	1.80
	Anakinra	41	1.57 ^a^	1.58	1.34 ^b^	1.91	0.96 ^c^	1.87
	*p* =		0.693		0.064		0.013	
Ferritin	Control Group	24	878.18 ^a^	749.52	832.09 ^a^	711.07	848.72 ^a^	883.93
	Anakinra	41	783.42 ^a^	740.26	597.65 ^b^	569.24	480.10 ^c^	422.31
	*p* =		0.532		0.097		0.054	
IL-6	Control Group	24	122.65 ^a^	303.71	62.99 ^a^	84.18	44.67 ^a^	37.06
	Anakinra	41	120.33 ^a^	184.10	34.90 ^b^	66.79	12.37 ^c^	22.64
	*p* =		0.876		<0.001		<0.001	
	Control Group	24	91.33 ^a^	3.62	91.71 ^a^	4.45	93.50 ^b^	5.38
Sat.O_2_	Anakinra	41	91.02 ^a^	4.13	92.80 ^b^	4.05	94.56 ^c^	5.20
	*p* =		0.784		0.204		0.034	

Identical letters across the horizontal rows indicate no statistically significant difference, while different letters indicate the presence of a statistically significant difference (*p* < 0.05). Changes are measured on Day 3 and Day 7, compared to the baseline values on Day 1. The grey color highlights the differences between measurements taken on Day 3 and Day 7 compared to the baseline values (both in absolute terms and percentages) where the dynamics are statistically significant. The yellow color indicates higher values in comparisons between male and female groups, as well as across different age groups.

The results showed a significant relationship between the dynamics of leukocytes, ASAT, LDH, D-dimer, ferritin, and IL-6, and the administration of Anakinra. A statistically significant decrease in ASAT and LDH values in the control group was noted only on Day 7. In the Anakinra group, significantly (or with borderline statistical significance, *p* < 0.1) lower mean values were observed for leukocytes, NLR, D-dimer, ferritin, and IL-6 on Days 3 and 7 compared to the control group. Additionally, significantly lower CRP values were detected in the Anakinra group on Day 7 compared to the control group. In our study, we observed that patients receiving Anakinra showed not only changes in the levels of laboratory indicators but also improvement regarding the oxygenation status. No statistically significant differences were found regarding the duration of hospitalization. Mortality rates were higher in the control group (12.50% versus 7.32% in the Anakinra group); however, due to the small sample size, no statistical difference was observed between the two groups. Further investigations with larger patient populations are needed to evaluate the potential benefits of Anakinra treatment on mortality rates.

The study has the following limitations: First, there is a small sample size and an imbalance between the control and Anakinra groups, as well as a gender imbalance. While uneven group sizes can introduce potential bias and reduce the robustness of statistical comparisons, we applied rigorous criteria consistently across both groups to minimize such effects. Additionally, we used statistical methods that are appropriate for smaller and unevenly distributed samples, reducing the risk of bias in dependency testing. Second, as a single-center study, the generalizability of our findings to broader populations may be limited. Third, the lack of long-term follow-up, partly due to poor patient compliance, restricts our ability to assess longer-term outcomes.

## 5. Conclusions

The experience from the SARS-CoV-2 pandemic, alongside the current state of infection spread and its global consequences, demonstrates that COVID-19 remains a significant public health issue and is likely to continue as such in the future. Despite its limitations, this study provides valuable insights into the dynamics of hyperinflammatory biomarkers in COVID-19 patients. The findings of this study underscore the need for future research into the therapeutic effects of Anakinra over a longer study period, not only concerning the biomarkers analyzed but also regarding its impact on clinical symptoms and long-term outcomes associated with post-COVID syndrome.

## Figures and Tables

**Table 1:** PCR result interpretation.

N Gene	E Gene	RdRP Gene	RNase P Gene (Internal Control)	Result
One of the three SARS-CoV-2 target genes is positive			+/−	Indeterminate
Two or three SARS-CoV-2 genes are positive			+/−	Positive
−	−	−	+	Negative
−	−	−	−	Invalid

**Table 2:** Characteristics of the groups.

Indicator		Anakinra Group	Control Group	*p*
Clinical form	Moderate	22	13	1.000
	Severe	19	11	
Hypertension and IHD	No	9	10	0.157
	Yes	32	14	
Diabetes	No	25	16	0.791
	Yes	16	8	
Chronic pulmonary disease	No	31	21	0.342
	Yes	10	3	
Alcohol usage	No	37	21	0.703
	Yes	4	3	
Smoking	No	6	4	1.000
	Yes	35	20	
Antibiotic treatment	No	1	0	0.382
	Ceftriaxone i.v.	35	24	
	Ceftriaxone + other i.v.	4	0	
	Cefuroxime p.o.	1	0	
Dexamethasone administration	No	23	11	0.452
	Yes	18	13	
28 survival rates	No	3	3	0.348
	Yes	38	21	

**Table 3:** Characteristics of the groups.

Indicator	Anakinra Group		Control Group		*p*
	$\bar{X}$	SD	$\bar{X}$	SD	
Duration of oxygen supplementation	2.10	2.58	3.29	3.68	0.226
Duration of hospitalization	7.76	1.28	8.21	1.38	0.134

## Data Availability

Data are contained within the article.

## References

[1] Wang D., Hu B., Hu C., Zhu F., Liu X., Zhang J., Wang B., Xiang H., Cheng Z., Xiong Y. (2020). Clinical Characteristics of 138 Hospitalized Patients with 2019 Novel Coronavirus-Infected Pneumonia in Wuhan, China. JAMA.

[2] Zhou F., Yu T., Du R., Fan G., Liu Y., Liu Z., Xiang J., Wang Y., Song B., Gu X. (2020). Clinical course and risk factors for mortality of adult inpatients with COVID-19 in Wuhan, China: A retrospective cohort study. Lancet.

[3] Felsenstein S., Herbert J.A., McNamara P.S., Hedrich C.M. (2020). COVID-19, Immunology and treatment options. Clin. Immunol..

[4] Hirano T., Murakami M. (2020). COVID-19: A New Virus, but a Familiar Receptor and Cytokine Release Syndrome. Immunity.

[5] Zhang C., Wu Z., Li J.W., Zhao HWang G.Q. (2020). The cytokine release syndrome (CRS) of severe COVID-19: Interleukin-6 receptor (IL-6R) antagonist Tocilizumab may be the key to reduce the mortality. Int. J. Antimicrob. Agents.

[6] Li C., He Q., Qian H., Liu J. (2021). Overview of the pathogenesis of COVID-19 (Review). Exp. Ther. Med..

[7] Chen G., Wu D., Guo W., Cao Y., Huang D., Wang H., Wang T., Zhang X., Chen H., Yu H. (2020). Clinical and immunological features of severe and moderate coronavirus disease 2019. J. Clin. Investig..

[8] Giamarellos-Bourboulis E.J., Netea M.G., Rovina N., Akinosoglou K., Antoniadou A., Antonakos N., Damoraki G., Gkavogianni T., Adami M.E., Katsaounou P. (2020). Complex Immune Dysregulation in COVID-19 Patients with Severe Respiratory Failure. Cell Host Microbe.

[9] Schulert G.S., Grom A.A. (2014). Macrophage activation syndrome and cytokine-directed therapies. Best Pract. Res. Clin. Rheumatol..

[10] Kyriazopoulou E., Leventogiannis K., Norrby-Teglund A., Dimopoulos G., Pantazi A., Orfanos S.E., Rovina N., Tsangaris I., Gkavogianni T., Botsa E. (2017). Macrophage activation-like syndrome: An immunological entity associated with rapid progression to death in sepsis. BMC Med..

[11] Halyabar O., Chang M.H., Schoettler M.L., Schwartz M.A., Baris E.H., Benson L.A., Biggs C.M., Gorman M., Lehmann L., Lo M.S. (2019). Calm in the midst of cytokine storm: A collaborative approach to the diagnosis and treatment of hemophagocytic lymphohistiocytosis and macrophage activation syndrome. Pediatr. Rheumatol..

[12] Vasbinder A., Padalia K., Pizzo I., Machado K., Catalan T., Presswalla F., Anderson E., Ismail A., Hutten C., Huang Y. (2024). SuPAR, biomarkers of inflammation, and severe outcomes in patients hospitalized for COVID-19: The International Study of Inflammation in COVID-19. J. Med. Virol..

[13] Liu Y., Zhang C., Huang F., Yang Y., Wang F., Yuan J., Zhang Z., Qin Y., Li X., Zhao D. (2020). Elevated plasma levels of selective cytokines in COVID-19 patients reflect viral load and lung injury. Natl. Sci. Rev..

[14] Chaudhary R., Garg J., Houghton D.E., Murad M.H., Kondur A., Chaudhary R., Wysokinski W.E., McBane R.D. (2021). Thromboinflammatory biomarkers in COVID-19: Systematic review and meta-analysis of 17,052 patients. Mayo Clin. Proc. Innov. Qual. Outcome.

[15] Henry B.M., de Oliveira M.H.S., Benoit S., Plebani M., Lippi G. (2020). Hematologic, biochemical and immune biomarker abnormalities associated with severe illness and mortality in coronavirus disease 2019 (COVID-19): A meta-analysis. Clin. Chem. Lab. Med..

[16] Wu C., Chen X., Cai Y., Xia J., Zhou X., Xu S., Huang H., Zhang L., Zhou X., Du C. (2020). Risk factors associated with acute respiratory distress syndrome and death in patients with coronavirus disease 2019 pneumonia in Wuhan, China. JAMA Intern. Med..

[17] Ling Y., Lin Y., Qian Z., Huang D., Zhang D., Li T., Liu M., Song S., Wang J., Zhang Y. (2020). Clinical analysis of risk factors for severe patients with novel coronavirus pneumonia. Chin. J. Infect. Dis..

[18] Elshazli R.M., Toraih E.A., Elgaml A., El-Mowafy M., El-Mesery M., Amin M.N., Hussein M.H., Killackey M.T., Fawzy M.S., Kandil E. (2020). Diagnostic and prognostic value of hematological and immunological markers in COVID-19 infection: A meta-analysis of 6320 patients. PLoS ONE.

[19] Cheng L., Li H., Li L., Liu C., Yan S., Chen H., Li Y. (2020). Ferritin in the coronavirus disease 2019 (COVID-19): A systematic review and meta-analysis. J. Clin. Lab. Anal..

[20] Short S.A.P., Gupta S., Brenner S.K., Hayek S.S., Srivastava A., Shaefi S., Singh H., Wu B., Bagchi A., Al-Samkari H. (2021). D-dimer and death in critically ill patients with coronavirus disease 2019. Crit. Care Med..

[21] Sproston N.R., Ashworth J.J. (2018). Role of C-Reactive protein at sites of inflammation and infection. Front. Immunol..

[22] Huang C., Wang Y., Li X., Ren L., Zhao J., Hu Y., Zhang L., Fan G., Xu J., Gu X. (2020). Clinical features of patients infected with 2019 novel coronavirus in Wuhan, China. Lancet.

[23] Liu J., Li S., Liu J., Liang B., Wang X., Wang H., Li W., Tong Q., Yi J., Zhao L. (2020). Longitudinal characteristics of lymphocyte responses and cytokine profiles in the peripheral blood of SARS-CoV-2 infected patients. EBioMedicine.

[24] Webb B.J., Peltan I.D., Jensen P., Hoda D., Hunter B., Silver A., Starr N., Buckel W., Grisel N., Hummel E. (2020). Clinical criteria for COVID-19-associated hyperinflammatory syndrome: A cohort study. Lancet Rheumatol..

[25] Yildirim M., Halacli B., Yuce D., Gunegul Y., Ersoy E.O., Topeli A. (2023). Assessment of Admission COVID-19 Associated Hyperinflammation Syndrome Score in Critically-Ill COVID-19 Patients. J. Intensive Care Med..

[26] Manson J.J., Crooks C., Naja M., Ledlie A., Goulden B., Liddle T., Khan E., Mehta P., Martin-Gutierrez L., Waddington K.E. (2020). COVID-19-associated hyperinflammation and escalation of patient care: A retrospective longitudinal cohort study. Lancet Rheumatol..

[27] Schneider M. (2022). The Role of Biomarkers in Hospitalized COVID-19 Patients with Systemic Manifestations. Biomark. Insights.

[28] Chen C.H., Lin S.W., Shen C.F., Hsieh K.S., Cheng C.M. (2022). Biomarkers during COVID-19: Mechanisms of Change and Implications for Patient Outcomes. Diagnostics.

[29] Khani E., Shahrabi M., Rezaei H., Pourkarim F., Afsharirad H., Solduzian M. (2022). Current evidence on the use of anakinra in COVID-19. Int. Immunopharmacol..

[30] European Medicines Agency (2021). EMA Recommends Approval for Use of Kineret in Adults with COVID-19.

[31] Nguyen T., Dima L., Willett K.C. (2023). Anakinra-An Interleukin-1 Receptor Antagonist for COVID-19. Am. J. Ther..

[32] Amikishiyev S., Durak G., Yilmaz A., Erelel M., Deniz R., Ince B., Çağatay A.A., Kose M., Gunver M.G., Bektas M. (2022). POS1216 Potential Predictors of Outcome for Anakinra Treatment in COVID-19 Patients with Macrophage Activation Syndrome. Ann. Rheum. Dis..

[33] Karakike E., Dalekos G.N., Koutsodimitropoulos I., Saridaki M., Pourzitaki C., Papathanakos G., Kotsaki A., Chalvatzis S., Dimakopoulou V., Vechlidis N. (2022). ESCAPE: An Open-Label Trial of Personalized Immunotherapy in Critically lll COVID-19 Patients. J. Innate Immun..

[34] Hussein A.A.M., Sayad R., Abdelshafi A., Hammam I.A., Kedwany A.M., Elkholy S.A.-E., Ibrahim I.H. (2023). A meta analysis on the utility of Anakinra in severe COVID-19 disease. Cytokine.

[35] Naveed Z., Sarwar M., Ali Z., Saeed D., Choudhry K., Sarfraz A., Sarfraz Z., Felix M., Cherrez-Ojeda I. (2022). Anakinra treatment efficacy in reduction of inflammatory biomarkers in COVID-19 patients: A meta-analysis. J. Clin. Lab. Anal..

[36] Potere N., DelBuono M.G., Caricchio R., Cremer P.C., Vecchie A., Porreca E., Gasperina D.D., Dentali F., Abbate A., Bonaventura A. (2022). Interleukin-1 and the NLRP3 inflammasome in COVID-19: Pathogenetic and therapeutic implications. EBioMedicine.

[37] Steinhardt M., Wiebecke S., Weismann D., Frantz S., Tony H., Klinker H., Schmalzing M. (2020). Biomarker-guided application of low-dose anakinra in an acute respiratory distress syndrome patient with severe COVID-19 and cytokine release syndrome. Scand. J. Rheumatol..

[38] (2015). Quality Management Systems–Requirements.

[39] (2016). Medical Devices – Quality Management Systems – Requirements for Regulatory Purposes.

[40] Ullah R., Khan J., Basharat N., Huo D., Din A.U., Wang G. (2022). Evaluation of Cardiac Biomarkers and Expression Analysis of IL-1, IL-6, IL-10, IL-17, and IL-25 among COVID-19 Patients from Pakistan. Viruses.

[41] Chen N., Zhou M., Dong X., Qu J., Gong F., Han Y., Qiu Y., Wang J., Liu Y., Wei Y. (2020). Epidemiological and clinical characteristics of 99 cases of 2019 novel coronavirus pneumonia in Wuhan, China: A descriptive study. Lancet.

[42] Tleyjeh I.M., Kashour Z., Riaz M., Hassett L., Veiga V.C., Kashour T. (2021). Efficacy and safety of tocilizumab in COVID-19 patients: A living systematic review and meta-analysis, first update. Clin. Microbiol. Infect..

[43] Kharazmi A.B., Moradi O., Haghighi M., Kouchek M., Manafi-Rasi A., Raoufi M., Shoaei D., Hadavand F., Nabavi M., Miri M.M. (2022). A randomized controlled clinical trial on efficacy and safety of anakinra in patients with severe COVID-19. Immun. Inflamm. Dis..

